# Experimental Evaluation of Food-Grade Semi-Refined Carrageenan Toxicity

**DOI:** 10.3390/ijms222011178

**Published:** 2021-10-16

**Authors:** Denys Pogozhykh, Yevgen Posokhov, Valeriy Myasoedov, Galina Gubina-Vakulyck, Tetyana Chumachenko, Oleksandr Knigavko, Hanna Polikarpova, Yuliia Kalashnyk-Vakulenko, Ketino Sharashydze, Oksana Nakonechna, Volodymyr Prokopyuk, Anatolii Onishchenko, Anton Tkachenko

**Affiliations:** 1 Clinic for Hematology, Hemostaseology, Oncology and Stem Cell Transplantation, Hannover Medical School, Carl-Neuberg-Str. 1, 30625 Hannover, Germany; 2 Research Institute of Experimental and Clinical Medicine, Kharkiv National Medical University, 6 Trinklera st, 61022 Kharkiv, Ukraine; yevgenposokhov@gmail.com (Y.P.); v.yu.prokopiuk@gmail.com (V.P.); ai.onishchenko@knmu.edu.ua (A.O.); 3 Department of Organic Chemistry, Biochemistry, Paints and Coatings, The National Technical University “Kharkiv Polytechnic Institute”, 2 Kyrpychova st, 61000 Kharkiv, Ukraine; 4 Department of Medical Biology, Kharkiv National Medical University, 4 Nauky ave, 61022 Kharkiv, Ukraine; vmyasoedov@ukr.net; 5 Department of Pathological Anatomy, Kharkiv National Medical University, 4 Nauky ave, 61022 Kharkiv, Ukraine; gvgipatology@gmail.com; 6 Department of Epidemiology, Kharkiv National Medical University, 12 Trinklera st, 61022 Kharkiv, Ukraine; tatalchum@gmail.com; 7 Department of Urology, Nephrology and Andrology, Kharkiv National Medical University, 195 Moskovsky ave, 61002 Kharkiv, Ukraine; ov.knihavko@knmu.edu.ua; 8 Department of Biochemistry, Kharkiv National Medical University, 4 Nauky ave, 61022 Kharkiv, Ukraine; hv.polikarpova@knmu.edu.ua (H.P.); oa.nakonechna@knmu.edu.ua (O.N.); 9 Department of Otorhinolaryngology, Kharkiv National Medical University, 4 Nauky ave, 61022 Kharkiv, Ukraine; ym.kalashnyk@knmu.edu.ua; 10 Department of Obstetrics and Gynecology, Kharkiv National Medical University, 4 Malinovskogo st, 61052 Kharkiv, Ukraine; keti74sh@gmail.com; 11 Department of Cryobiology of the Reproductive System, Institute for Problems of Cryobiology and Cryomedicine of the National Academy of Sciences of Ukraine, 23 Pereyaslavskaya st, 61015 Kharkiv, Ukraine

**Keywords:** carrageenan, E407a, processed *Eucheuma* seaweed, toxicity, fluorescent probe, leukocytes, inflammation, intestine, animal model

## Abstract

The safety of food additives E407 and E407a has raised concerns in the scientific community. Thus, this study aims to assess the local and systemic toxic effects of the common food additive E407a in rats orally exposed to it for two weeks. Complex evaluations of the effects of semi-refined carrageenan (E407a) on rats upon oral exposure were performed. Local effects of E407a on the intestine were analyzed using routine histological stains and CD68 immunostaining. Furthermore, circulating levels of inflammatory markers were assessed. A fluorescent probe O1O (2- (2′-OH-phenyl)-5-phenyl-1,3-oxazole) was used for evaluating the state of leukocyte cell membranes. Cell death modes of leukocytes were analyzed by flow cytometry using Annexin V and 7-aminoactinomycin D staining. Oral administration of the common food additive E407a was found to be associated with altered small and large intestinal morphology, infiltration of the lamina propria in the small intestine with macrophages (CD68^+^ cells), high systemic levels of inflammation markers, and changes in the lipid order of the phospholipid bilayer in the cell membranes of leukocytes, alongside the activation of their apoptosis. Our findings suggest that oral exposure to E407a through rats results in the development of intestinal inflammation.

## 1. Introduction

Marine polysaccharides commercially extracted from seaweeds have found wide application in the food, biomedical, and pharmaceutical industries [1]. At the moment, algae-derived carbohydrates such as carrageenans (CGNs), fucoidans, laminarins, and alginates are considered to be promising antivirals, immunomodulators, anticoagulants and wound-healing agents, drug delivery tools, etc. [2,3,4,5,6]. Due to oceanic biodiversity, the field of naturally occurring marine polymers is currently expanding, with more studies focusing on the physicochemical characterization of algal carbohydrates and the exploration of their significance, as well as their potential application in biomedicine. CGNs are among the most investigated of all marine polysaccharides and are believed to have high biomedical potential. CGNs are negatively charged, unbranched, sulfated glycans isolated from red algae, including *Eucheuma, Gigartina*, and *Chondrus crispus* [7]. Structurally, they are similar to human glycosaminoglycans and consist of repeating disaccharide units composed of D-galactose derivatives [3,8]. The type of bonds that link monosaccharide units determines the form of CGNs. The major forms are λ (lambda), κ (kappa), and ι (iota) with prevalence of the κ type, which accounts for 60% of all CGNs [5].

CGNs are widely used as food additives. They are registered as E407 (refined CGN) and E407a (semi-refined CGN or processed *Eucheuma* seaweed, also known as PES). The history of this food additive dates back to the beginning of the 19th century when its potential as a gelling agent was recognized. The hydrocolloid properties of CGNs promoted their application in the food industry as thickeners and texturizers. It is worth mentioning that native CGN is used in the production of processed meat, dairy products, jellies, and confectionary. Meanwhile, PES is added to processed meat to improve its value [9]. Moreover, more cost-effective CGNs are used to replace food components in meat and dairy products. Such food fraud has become more common [10]. It was reported that the average daily consumption of CGNs in the Western diet may reach 2.8 g for a 70 kg individual, i.e., up to 40 mg/kg of weight per day [11].

In recent years, contradictory reports concerning the safety of CGNs have emerged. A University of Illinois research team claimed that CGNs are unsafe for oral consumption and induce inflammation in the gut [12,13]. However, other toxicological studies have found no evidence that CGNs may upregulate pro-inflammatory molecules and promote intestinal inflammation [14]. Of note, data on the safety of CGNs obtained in cell culture experiments should not be overinterpreted as CGN and its interaction in the gut with colonic microbiome are poorly understood [15,16,17,18]. Yet, this is of great importance due to recent reports on the ability of CGNs to magnify pathogen-induced inflammation in the intestine of mice [19], contribute to inflammatory bowel disease aggravation [20,21], and enhance lipopolysaccharide (LPS)-induced production of pro-inflammatory cytokines such as tumor necrosis factor-alpha (TNFα) and interleukin-8 (IL-8) [19,22]. Thus, both in vivo and in vitro studies are further required to assess the safety of CGNs.

The aim of our research is to assess the possible local and systemic toxic effects in rats orally exposed to the common food additive E407a.

## 2. Results

The morphological status of both small and large intestines in animals from the control and experimental groups was analyzed to assess the ability of E407a to induce inflammation.

**Figure 1:**
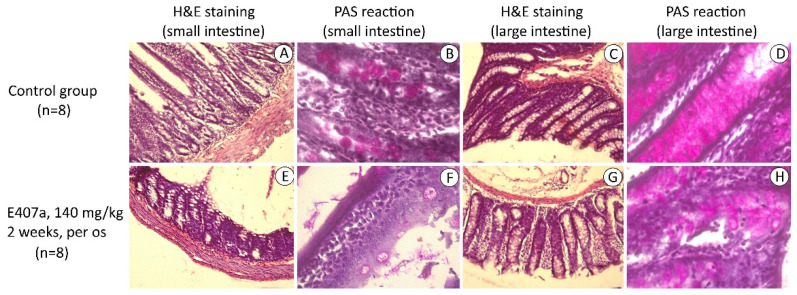
Representative hematoxylin-eosin (H&E)-stained and periodic acid-Schiff (PAS)-stained sections of the small and large intestines and PAS stain. (**A**) Control group. Small intestine is not damaged. Villi are preserved. H&E stain, 100×. (**B**) Control group. Moderately PAS-positive mucin-containing goblet cells are visible. PAS stain, 400×. (**C**) Control group. No signs of inflammation in the large intestine. The mucosal layer is intact. H&E stain, 400×. (**D**) Control group. PAS stain reveals goblet cells with mucin inside. PAS stain, 400×. (**E**) Experimental group. Villi are absent. Significant leukocyte infiltration is observed. H&E stain, 100×. (**F**) Experimental group. Multi-row epithelia is noticed. Goblet cells contain less PAS-positive mucin. PAS stain, 400×. (**G**) Experimental group. Epithelium is desquamated. Leukocyte infiltration is observed. H&E stain, 100×. (**H**) Experimental group. PAS positivity of mucin in goblet cells is less pronounced compared with the control group. PAS stain, 400×.

The small intestinal morphology in control animals was not altered. The villi exhibited its typical shape, epithelial cells were preserved, and no evidence of inflammation was observed (Figure 1).

However, the histological evaluation revealed damage in the small intestinal mucosa in the experimental group. A number of villi appeared deformed and destroyed. Furthermore, the villi were de-epithelialized. The lamina propria was characterized by a noticeable infiltration with both macrophages and lymphocytes (Figure 1). The amount of goblet cells in the small intestine was reduced (Figure 1).

In control animals, the colonic mucosal layer was well-preserved. The superficial epithelia were represented by both cylindrical and goblet cells. The cells were not damaged. The lamina propria was thin and slightly infiltrated with leukocytes (Figure 1). Regions with more pronounced infiltration were only noted in the lower portions of the mucosa. Mucin-secreting columnar goblet cells in the crypts were filled with moderate fuchsinophillic mucin (Figure 1).

Microscopic analysis revealed overexpansion of the large intestine in the experimental group. As a result, the mucosal layer was thinner. It is noteworthy that the superficial epithelia were desquamated in some regions (Figure 1). Crypts were covered with goblet cells whose numbers diminished. The cells were less fuchsinophillic in comparison to the control group (Figure 1). The lamina propria was thicker in the entire mucosal layer and noticeably infiltrated with macrophages and lymphocytes. Eosinophils were frequently found. The epithelial lining of crypts also contained more leukocytes. The lamina propria and submucosal layer adjacent to the muscular layer were thicker and more infiltrated in comparison with controls.

**Figure 2:**
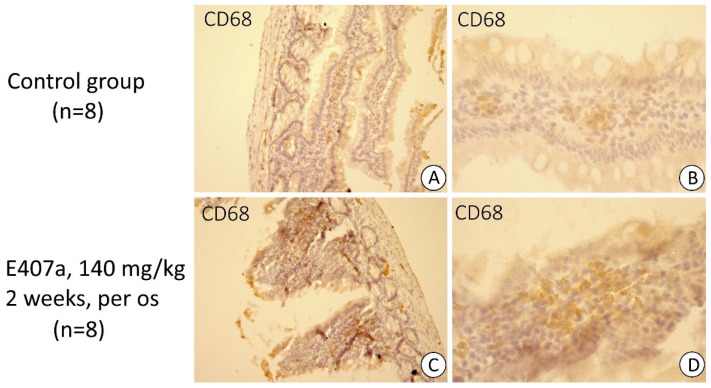
Immunostaining for CD68 (brown stain) in the small intestine of control animals ((**A**), 100×; (**B**), 400×) and rats treated with E407a for two weeks ((**C**), 100×; (**D**), 400×). Moderate amounts of CD68^+^ cells (macrophages) are observed in control samples. Exposure to E407a results in an increase in the number of CD68^+^ cells in the lamina propria.

**Figure 3:**
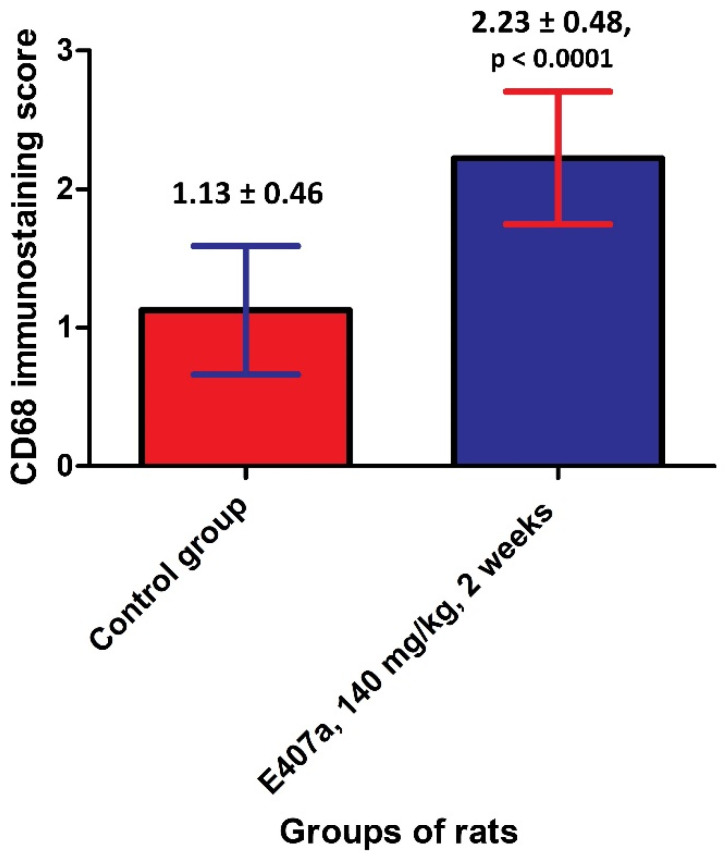
Quantitative analysis of CD68 expression in the small intestine reveals an infiltration of the small intestinal lamina propria with CD68^+^ cells in rats orally exposed to E407a.

Visually, the amount of CD68 positively stained cells, i.e., macrophages, was higher in the small intestinal stroma in the experimental group compared with controls. The cells tended to form groups consisting of several macrophages in the lamina propria (Figure 2). It is noteworthy that CD68^+^ cells mainly accumulated in the regions with the most pronounced damage to epithelia. The data of immune-scoring supplemented this observation, confirming a higher number of CD68-expressing cells in the lamina propria of rats orally exposed to E407a (Figure 3).

Morphological studies were complemented by the determination of inflammation markers in blood serum. Oral exposure to E407a resulted in a 7.4-fold elevation of circulating C-reactive protein (CRP) and a 5.3-fold increase in the content of middle molecules (MMs) in blood serum compared with the control group (Table 1).

**Figure 4:**
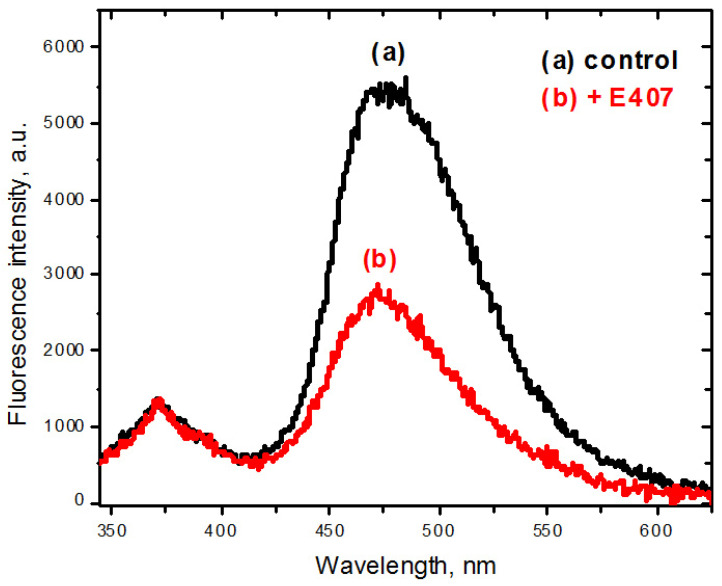
Representative fluorescence spectra of probe O1O in leukocyte suspensions: (**a**) the control group of rats (black solid line); (**b**) the animals orally exposed to E407a for two weeks (red solid line). For a correct comparison, the spectra were normalized to the fluorescence intensity of the normal form.

The results of the fluorescence measurements are presented in Figure 4 and Table 2.

A short-wavelength shift (~5 nm) of the fluorescence maximum of the photo-tautomer (T *) was observed in the fluorescence spectrum for the experimental group of animals in comparison with the corresponding spectrum for the control sample (Figure 4). The mentioned short-wavelength shift indicates an increase in polarity of the probe microenvironment [23,24,25] in leukocyte membranes after E407a administration. 

Furthermore, a decrease in fluorescence intensity of the photo-tautomer (Figure 4, see emission: 420–600 nm) and, hence, a decrease in the ratio of the fluorescence intensities of the photo-tautomer and normal forms (I_T_ */I_N_ *) of probe O1O (Table 2), were observed for the leukocyte membranes in leukocytes of the experimental group of rats after oral exposure to E407a for two weeks in comparison with the corresponding parameters of the control group. The decrease in the fluorescence intensity ratio (I_T_ */I_N_ *) points to both the increase in polarity [23,24,25] and the increase in the proton-donor ability of the probe O1O [26] environment in the leukocyte membranes of rats orally exposed to E407a.

**Figure 5:**
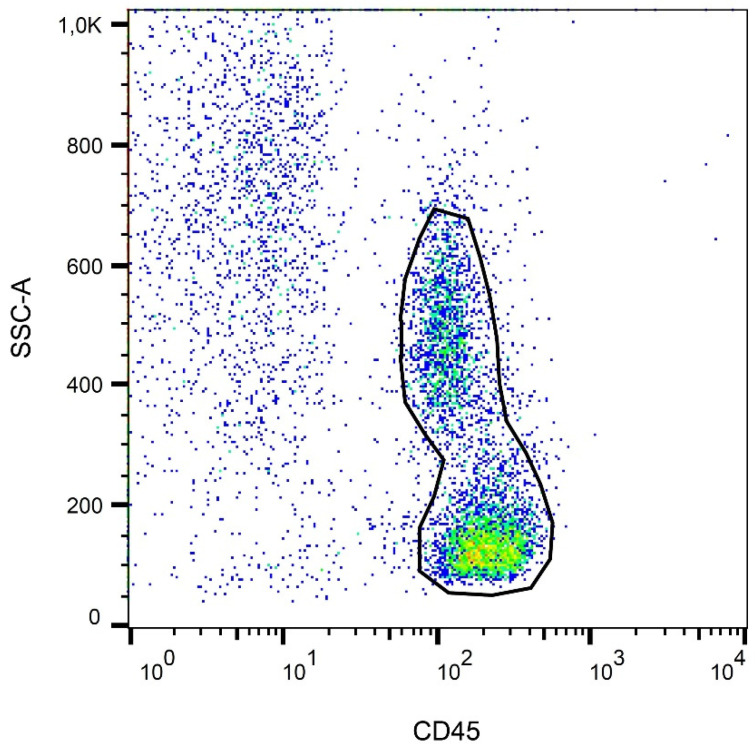
Representative SSC/APC-CyTM 7 CD45 demonstrating the gating strategy for identifying CD45^+^ cells.

**Figure 6:**
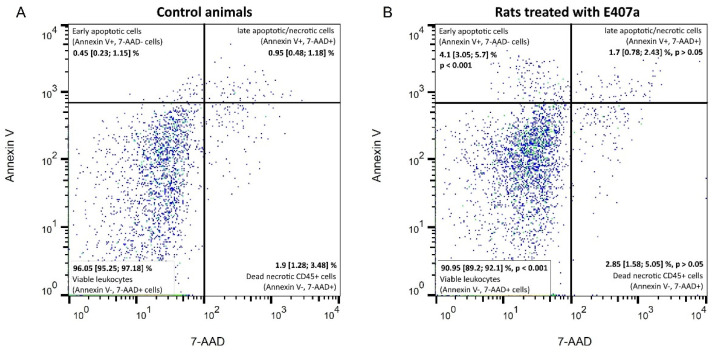
Representative Annexin V/7-AAD dotplots that demonstrate the percentage (Me [IQR]) of viable leukocytes (Annexin V^−^, 7-AAD^+^ cells); early apoptotic cells (Annexin V^+^, 7-AAD^−^ cells); late apoptotic/necrotic cells (Annexin V^+^, 7-AAD^+^); and dead necrotic CD45^+^ cells (Annexin V^−^, 7-AAD^+^) in the control (**A**) and experimental groups (**B**). A statistically significant increase in the percentage of early apoptotic cells is found in rats exposed to semi-refined carrageenan.

Analysis of viability and cell death modes of leukocytes was performed on CD45^+^ cells. The gating strategy is shown in Figure 5. Numerical data on the percentages of viable and apoptotic leukocytes are presented in Figure 6. A statistically significant difference (*p* < 0.001) in the amount of Annexin V^−^, 7-AAD^−^ viable CD45^+^ cells between the studied groups was observed. Moreover, oral exposure to E407a for two weeks was associated with a nine-fold increase in the percentage of Annexin V^+^, 7-AAD^−^ early apoptotic white blood cells. At the same time, no statistically significant differences (*p* > 0.05) were found in the percentages of either Annexin V^+^, 7-AAD^+^ late apoptotic/necrotic leukocytes or Annexin V^−^, 7-AAD^+^ dead necrotic cells (Figure 6).

## 3. Discussion

There is a growing body of evidence that CGNs promote inflammation in experimental animals and cell-based experiments [1,12,13,27,28,29,30]. In addition, carrageenans are known to enhance LPS-induced TNF-α production in leukocytes. It is important to mention that carrageenans have no impact on TNF-α expression in the absence of LPS [22]. Furthermore, stimulatory effects of carrageenans on LPS-induced inflammation are found to be mediated by the bcl-10-NF-kB pathway [19]. Our data appear to be consistent with reports on the pro-inflammatory effects of carrageenans. In this study, the oral intake of semi-refined carrageenans by rats was associated with altered intestinal morphology in the small and large intestines, as well as infiltration of the small intestinal mucosa with macrophages. These findings indicate the development of local inflammation in response to E407a. Histological and immunostaining data are supplemented by analysis of circulating concentrations of CRP and MMs (markers of inflammation and endogenous intoxication) presented in this study. However, reports on CGN toxicity are harshly criticized [31], indicating that high-quality experimental studies have demonstrated no CGN toxicity [32,33]. It is worth noting that criticism is primarily based on the assumption that results of studies are misinterpreted due to the confusion of terms. In particular, low-molecular-weight CGNs, such as poligeenan and degraded CGN, are used in experiments instead of high-molecular-weight food-grade CGNs. The former are well-characterized toxic substances and their use in the food industry is prohibited [34]. However, in this study, the food-grade CGN was used as a component of the food additive E407a, which is generally recognized as safe by the Food and Drug Administration. 

In addition to the general safety of CGN, there is controversy concerning its fate in the alimentary tract [35]. It is not clear whether CGN is digested or not. There is evidence that CGN may partially undergo acid hydrolysis in the stomach [36]. However, such data are scarce and controversial. Most studies indicate that CGNs are neither absorbed nor degraded in the gut [31]. However, in the present study, our findings are not limited to the intestine. Systemic effects include changes in the lipid order of leukocyte cell membranes and the activation of their apoptosis.

The increase in the polarity and the proton-donor ability of the microenvironment of probe O1O points to increased hydration [37,38,39,40,41] of the region of the probe location: in the area of glycerol backbones of phospholipids, closer to the center of the lipid bilayer; in the area of carbonyl groups of phospholipids; in the area of hydrocarbon chains of phospholipids; and near the carbonyl groups of phospholipids. In turn, such higher hydration suggests a reduced membrane lipid order (i.e., higher membrane fluidity) [30,37,41]. Thus, our findings provide evidence that the oral intake of E407a leads to a decrease in the lipid order (i.e., to the increase in fluidity) of rats’ leukocyte membranes. Such changes in the lipid order of cell membranes may be associated with apoptosis. The reduction in lipid order was reported in apoptosis induced by different factors [42]. Furthermore, Pyrshev K.A. et al. [43] stated that the activation of caspase-3, a key executioner protease in both intrinsic and extrinsic apoptotic pathways, correlates with the reduction in lipid order in the outer leaflet of cell membranes, and the decreased lipid order of cell membranes can be used to detect early apoptosis. Thus, the next step of this study was to analyze the rate of apoptosis in rats exposed to semi-refined carrageenans. Obtained flow-cytometric data clearly indicate the activation of early apoptosis in animals administered with E407a, which is consistent with the findings on reduced lipid order in membranes of leukocytes. Another study also demonstrated that the oral intake of the refined carrageenan (E407) could lead to the activation of apoptosis in leukocytes [44]. Furthermore, the authors showed the activation of leukocyte necrosis, which, however, was not observed in our study. Yet, it is worth mentioning that cells directly treated with carrageenans do not undergo apoptosis and their viability is not affected [14,45], suggesting another mechanism of apoptosis activation in response to its oral consumption. Such extraintestinal changes described herein and developed in response to E407a oral intake can be mediated by pro-inflammatory cytokines [46].

Considering the fact that food-grade CGN is mixed with luminal contents, it is important to evaluate the interplay between ingested CGNs and intestinal microbiota, especially in light of recent data [19] on the ability of CGNs to magnify LPS-induced inflammation in the gut. This is of great concern for compromised populations, and in particular for patients with inflammatory bowel disease (IBD). This assumption is supported by recent reports on the ability of CGN supplementations to contribute to relapse in patients with ulcerative colitis [47]. In addition, the lost intestinal barrier integrity in IBD may contribute to higher absorptions of CGN.

Therefore, further studies are required to highlight gaps in knowledge on the CGN safety profile, its presence in the gut, and features of its interaction with the intestinal microbiota.

## 4. Materials and Methods

### 4.1. Animals and Groups

A total of 16 adult WAG rats were used in the study. They were randomly divided into two groups (*n* = 8 in each) after acclimatization in the vivarium for at least 2 weeks before the experiment. The animals from the experimental group were orally administered 1% PES solution in drinking water (140 mg of E407a per kg of weight on a daily basis) for 2 weeks, daily. The control rats were fed on a standard diet. Water was provided ad libitum.

### 4.2. Morphological and Immunohistochemical Studies

Samples of both small and large intestines were collected from rats of control and experimental groups. Formalin-fixed, paraffin-embedded sections were prepared from the corresponding organs. Sections were sliced into 4 μm thick microslides using a microtome. The slides were stained with hematoxylin and eosin solutions to assess the morphology of the intestine. Periodic acid-Schiff staining was performed for the evaluation of mucin formation. 

Small intestinal samples obtained from animals of both groups were stained with antibodies to rat CD68 pan-macrophage cell markers. Briefly, after deparaffinization with xylene and a PBS wash, the sections were treated with a hydrogen peroxide block and incubated with the primary antibodies to rat CD68 (Agilent Technologies, Santa Clara, CA, USA). Afterward, the samples were washed with PBS twice. Visualization of stained CD68 markers was performed using the Epredia™ UltraVision™ Quanto HRP DAB (Thermo Fischer Scientific, Waltham, MA, USA).

Stained microslides were analyzed microscopically with the application of the Axiostar Plus microscope (Zeiss, Oberkochen , Germany).

Expression of CD68 was evaluated quantitatively using a 0–3 scale. The number of CD68-displaying cells was counted per 1 mm^2^ in the regions of small intestinal lamina propria. Five areas (400×) were analyzed in each sample. The score was assigned based on the CD68-positive cells/CD68-negative cells ratio. A score of 0 indicated that the percentage of CD68-expressing cells was 10% and below. Scores of 1, 2, and 3 corresponded to 11–30%, 31–60%, and over 61%, respectively [48].

### 4.3. Determination of Inflammatory Markers in Blood Serum

Circulating levels of acute-phase protein C-reactive protein (CRP) and middle molecules (MMs), as a biomarker of endogenous intoxication, were analyzed to assess the presence of inflammation. CRP concentrations were determined by commercially available kits (Filicit-Diagnostika, Dnipro, Ukraine). Levels of MMs in serum were evaluated using the Gabrielyan method [49]. Briefly, trichloroacetic acid was added to blood serum samples. After centrifugation for 20 min at 1700× *g*, the samples were diluted with distilled water (1:10, *v/v*). The measurement was carried out on Stat Fax 1904 Chemistry Analyzer (Awareness Technology, Palm City, FL, USA) at λ = 254 nm and λ = 280 nm. The absorbance ratio of 280-to-254 nm was calculated. 

### 4.4. Lysis Protocol for Analysis of Fluorescent Probes and Flow Cytometry

To perform both the steady-state fluorescence spectroscopy and flow cytometry analysis, the leukocyte suspensions were obtained from the blood of rats of experimental and control groups with the application of the lyse/wash procedure (Becton Dickinson Technical Support Protocol, 2002). To isolate white blood cells (WBCs), 100 μL of blood from each rat was added to 12 × 75 mm capped polystyrene test tubes with the subsequent addition of 2 mL of 1× PharmLyse solution (BD, Franklin Lakes, NJ, USA). The solutions were incubated for 15 min at room temperature in the dark and centrifuged at 500× *g* for 5 min to lyse erythrocytes. The supernatant was discarded, followed by adding 2 mL of phosphate-buffered saline (PBS, pH 7.4; BD, Franklin Lakes, NJ, USA). Centrifugation was performed at 500× *g* for 5 min. The supernatant was discarded and 1 mL of PBS was added. This washing procedure for leukocytes was performed twice. Obtained WBC suspensions were used for the incubation with the fluorescent probes O1O (2- (2′-OH-phenyl)-5-phenyl-1,3-oxazole).

### 4.5. Analysis of Leukocyte Viability and Cell Death Modes

The viability of leukocytes and cell death modes were analyzed with flow cytometry. After double washing, leukocyte pellets were resuspended in 1 mL of 1× binding buffer. Then, 100 μL of suspensions were transferred to 12 × 75 mm capped polystyrene test tubes with the subsequent addition of 10 μL APC-CyTM 7 mouse anti-rat CD45 (BD, Franklin Lakes, NJ, USA), 5 μL FITC Annexin V, and 5 μL 7-AAD (BD, Franklin Lakes, NJ, USA). The suspensions were gently vortexed and incubated for 15 min in the dark at room temperature. Then 400 µL of 1× binding buffer was added to each test tube. Flow cytometry was performed with a FACSCanto™ II flow cytometry system (BD, Franklin Lakes, NJ, USA) counting 10,000 events per measurement. 

FlowJo 10.7.2 software (BD, Franklin Lakes, NJ, USA) was used to assess the flow cytometry results. Initially, the region of CD45^+^ cells was gated. Then, Annexin V and 7-AAD stained CD45^+^ cells were analyzed. This staining is used to identify four possible states of leukocytes: 1, viable leukocytes (Annexin V^−^, 7-AAD^+^ cells); 2, early apoptotic cells (Annexin V^+^, 7-AAD^−^ cells); 3, late apoptotic/necrotic cells (Annexin V^+^, 7-AAD^+^); 4, dead necrotic cells (Annexin V^−^, 7-AAD^+^).

### 4.6. Characteristics of the Fluorescent Probe

The probe was dissolved in acetonitrile to the initial concentration of ~2 × 10^–4^ mol/L (stock solution). An aliquot of the probe stock solution was added to the WBC suspensions to a final probe concentration of ~5 × 10^–6^ mol/L. The lipid-to-probe molar ratio was ~200:1. The cell suspensions were incubated with the probe for 1 h at room temperature prior to measurement. A 10 × 10 mm cuvette was used for the measurements. The fluorescence spectra were recorded on a Thermo Scientific Lumina fluorescence spectrometer (Thermo Fisher Scientific, Waltham, MA, USA) within the range of 350–630 nm, with an increment of 0.1 nm. Data were collected with 0.02 s intervals. The slits on the excitation and emission monochromators were 5 and 10 nm. The excitation wavelength was 330 nm.

A fluorescent probe O1O (2- (2′-OH-phenyl)-5-phenyl-1,3-oxazole) was used in this study, due to the dependence of its fluorescence parameters on the physico-chemical properties of its microenvironment: the proton-donor ability, the polarity, and viscosity of the microenvironment [23,24,25,50].

**Figure 7:**
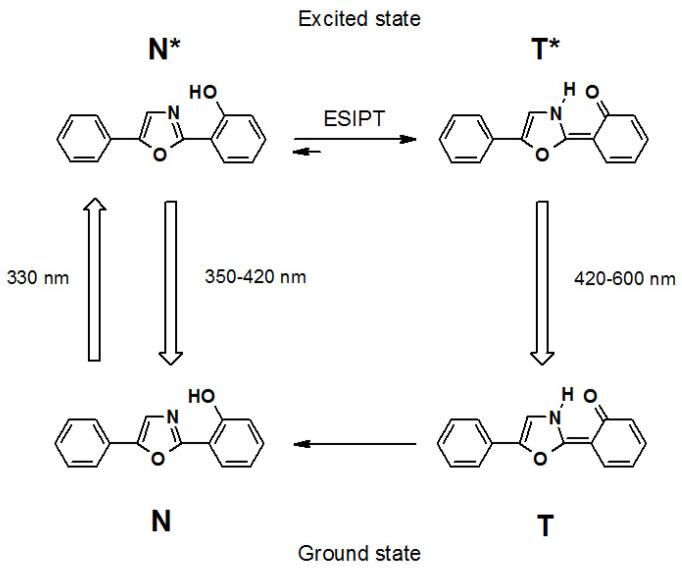
Scheme of excited state intramolecular proton transfer (ESIPT) reaction in 2 (2′-hydroxyphenyl)-5-phenyl-1,3-oxazole (probe O1O). The upwards arrow denotes the electronic excitation and the downwards arrow designates the emission of light (fluorescence). Corresponding maximum absorption and the ranges of emission are shown in nanometers. (Modified from Posokhov and Kyrychenko, 2018).

When the ortho-hydroxy 2,5-diaryl-1,3-oxazole is in the excited state, the excited-state proton transfer (ESIPT) reaction occurs [23,24,49,50]: the hydroxyl group in the ortho-position of the lateral benzene ring acts as a proton donor, whereas the nitrogen atom of the oxazole ring acts as the proton acceptor (Figure 7). This reaction results in the formation of the photoproduct (photo-tautomer form (T *)). In comparison with the initial (or alleged “normal”) form (N *), the photo-tautomer form (T *) is fluorescent in significantly longer wavelengths [23,24,25,50]. 

The presence of two-band fluorescence enables the ratiometric measurement, i.e., to use the ratio of the photo-tautomer form and the initial form fluorescence intensities (I_T_ */I_N_ *) as a parameter for estimating the physical and chemical properties of the microenvironment.

The usage of ratiometric fluorescent probes eliminates the measurement error caused by the deviation of the fluorescent probe concentration (e.g., uneven content of fluorescent probe in various membranes) and the measurement errors due to the deviation in configuration and adjustment of equipment for measurements of fluorescence (e.g., changes in the sensitivity of the photodetector, deviation in the intensity of the source of excitation light, etc.) [51,52].

**Figure 8:**
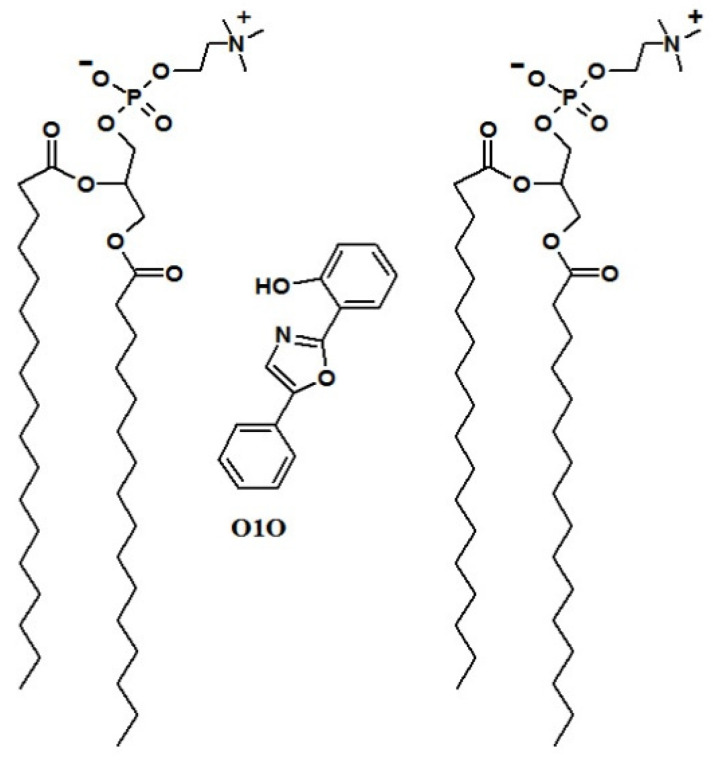
Localization and orientation of fluorescent probe O1O (2- (2′-OH-phenyl)-5-phenyl-1,3-oxazole) in phospholipid membranes. Two molecules of phosphatidylcholine from the outer leaflet are shown to denote the localization of the probe. (Adapted from Posokhov and Kyrychenko, 2018).

The localization and orientation of probes O1O (2- (2’-OH-phenyl)-5-phenyl-1,3-oxazole) in the cell membrane are presented in Figure 8. Probe O1O is localized in the area of the glycerol backbones of phospholipids (closer to the center of the lipid bilayer), in the area of carbonyl groups of phospholipids, and in the area of hydrocarbon chains of phospholipids (near the carbonyl groups of phospholipids).

The localization and orientation of probe O1O in the cell membrane are proposed on the basis of their fluorescent properties in lipid membranes [23,26], calculations of their location using a method of molecular dynamics [26], and their structural similarity to the fluorescent probes with a known location and orientation in lipid membranes [53].

### 4.7. Statistical Analysis

Comparisons between two independent groups of variables were performed using a non-parametric Mann–Whitney U test. Results are represented as medians and interquartile ranges. When the *p*-value was below 0.05, the data were assumed statistically significant. Statistical analyses were carried out with GraphPad Prism 5.0 software (GraphPad Software, USA).

## 5. Conclusions

Our findings suggest that the oral intake of the food-grade carrageenan-containing food additive E407a results in the development of intestinal inflammation manifested by both tissue and systemic effects.

## Figures and Tables

**Table 1:** Circulating inflammatory biomarkers in rats orally exposed to the common food additive E407a (Me [IQR]).

Groups of Animals	Control Group (*n* = 8)	Experimental Group (*n* = 8)	*p* Value
Parameters (Units)			
C-reactive protein (mg/L)	1.10 [1.01; 1.20]	8.10 [6.87; 9.55]	0.0002
Middle molecules (standard units)	0.086 [0.077; 0.090]	0.457 [0.387; 0.536]	0.0009

**Table 2:** The ratio of the fluorescence intensities of the photo-tautomer and normal forms (I_T_ */I_N_ *) of probe O1O in the leukocyte membranes of rats orally exposed to E407a (Me [IQR]).

Groups of Animals	I_T_ */I_N_ * (I_477_/I_371_)
Control group	4.1 [3.3; 4.7]
Rats orally exposed to E407a	2.0 [1.6; 2.3] *p* < 0.001

## Data Availability

All relevant data are contained within the article.
